# Efficacy of Preoperative Imaging Features and Blood Tests in Predicting the Increased Risk of Conversion in Laparoscopic Appendectomy Surgery

**DOI:** 10.7759/cureus.17092

**Published:** 2021-08-11

**Authors:** Banu Yigit, Esma Cerekci, Yasin Cakir, Bulent Citgez

**Affiliations:** 1 General Surgery, Elazig Fethi Sekin City Hospital, Elazig, TUR; 2 Radiology, Sisli Hamidiye Etfal Medical Practice and Research Center, University of Health Sciences, Istanbul, TUR; 3 General Surgery, Sisli Hamidiye Etfal Medical Practice and Research Center, University of Health Sciences, Istanbul, TUR

**Keywords:** appendicitis, conversion, blood tests, laparoscopic appendectomy, ultrasonography

## Abstract

Background

In this study, we identified preoperative risk factors, including imaging features and blood tests, to predict conversion from laparoscopic appendectomy to open appendectomy. Thus, we aimed to prevent patients from being exposed to the risks of laparoscopy by choosing patients for whom proceeding directly to an open surgery as an initial approach was appropriate.

Patients and methods

The cohort of 632 patients who underwent laparoscopic appendectomy due to acute appendicitis in our center between January 2017 and March 2021 were analyzed, and 521 of these patients comprised the study population. Baseline characteristics, medical history, preoperative laboratory tests, imaging features, and postoperative pathologic findings of all patients according to groups who underwent laparoscopic appendectomy or conversion to open appendectomy were examined.

Results

Among 521 patients, the appendectomy procedure was completed laparoscopically in 498 (95.6%) patients, and conversion to open appendectomy was occurred in 23 (4.4%) patients. 223 (42.8%) patients were female, and 298 (57.2%) patients were male. The mean age of all patients was 35.17±12.61 years (range, 16-80 years). Preoperative ultrasonography feature associated with a higher rate of conversion was free fluid collection (p=0.001). The levels of C-reactive protein, neutrophil, and neutrophil/lymphocyte ratio on admission were found to be significantly higher in the conversion group compared to the laparoscopy group (p=0.001, p=0.027, p=0.02, respectively).

Conclusions

Free fluid collection detected by ultrasonography, and elevation of C-reactive protein, neutrophil, and neutrophil/lymphocyte ratio may be useful in the prediction of a high risk of conversion appendectomy. Despite the unquestionable advantages of laparoscopic surgery, there are still substantial conversion rates. Within this framework, our study will help the surgeons to choose the most appropriate surgical methods for patients by evaluating them individually, and to inform them of the possibility of conversion to the open approach, and other risks before surgery.

## Introduction

The lifetime risk of acute appendicitis in an adult population ranges from 7% to 8%, and 10% of the patients having symptoms of acute appendicitis usually resulting in the removal of a normal appendix [1, 2]. Laparoscopic appendectomy (LA) deserves to be a gold standard, and has been widely accepted in acute appendicitis with decreased postoperative ache, short postoperative fasting period, rapid duration of recovery, shorter hospital stay, early resumption of normal activity, and superior cosmetic outcomes [3]. The decision to convert from laparoscopy to the open approach usually depends on preoperative parameters, skills of the surgeon, technical troubles or intraoperative complications, including bleeding, iatrogenic bowel injuries, and other complications. The conversion frequencies reported in the literature are variable, and range between 0% and 47% [4]. The conversion from laparoscopy to open surgery means perioperative adverse outcomes such as increased blood loss, additional incision, lengthened operative time, and postoperative adverse outcomes such as more analgesic requirement, delayed oral intake, wound infection, lengthened hospitalization time, and increased costs. Several studies were conducted to explain the predictor factors for conversion appendectomy (CA), including dense adhesions, diffuse peritonitis, and difficulties in excision of the appendix due to perforation or severe inflammation, but no algorithm has been developed so far [5-7].

In the present study, we assessed if there is a relationship between preoperative laboratory findings and imaging features and the risk of conversion to open appendectomy (OA) during LA. Thus, we aimed to select patients who could benefit from OA instead of laparoscopic surgery as an initial approach.

## Materials and methods

The data of 1662 cases who underwent appendectomy in Sisli Hamidiye Etfal Training and Research Hospital between January 2017 and March 2021, were analyzed. 632 patients were attempted LA, and 1030 patients underwent primary OA for acute appendicitis. Of the 632 attempted LAs, 521 met the inclusion criteria; 498 (95.6%) were completed successfully, and 23 (4.4%) were converted to open surgery. This retrospective study was approved by the Clinical Research Ethics Committee of University of Health Sciences, Sisli Hamidiye Etfal Research and Training Hospital (approval number: 3317; approval date: 08/06/2021).

We divided patients into two groups.

Group 1: Appendectomy wasn't completed laparoscopically, and the surgeon converted to an open surgery. 

Group 2: Appendectomy was completed laparoscopically.

Complete physical examination of the abdomen was performed for every patient. Blood tests and preoperative imaging tests, including ultrasonography (US) and/or computed tomography (CT), were ordered following a physical examination. Age, sex, preoperative laboratory tests, body mass index (BMI), time since first complaint, dominant presenting symptoms, the mean hospital stay, imaging features, and pathological findings were recorded.

Inclusion criteria

We included patients who were diagnosed with acute appendicitis and in whom we performed LA as an initial surgical approach.

Exclusion criteria

Electively operated cases, negative appendectomy cases, incidental appendectomy cases, patients who had undergone previous lower abdominal surgery, and conservatively treated patients were excluded from the study. Also, the patients who had incomplete records of clinical data or insufficient follow-up were excluded from the study.

Laboratory tests

The complete blood count was also obtained from both groups, and white blood cell (WBC), neutrophil, lymphocyte, neutrophil/lymphocyte ratio (NLR), red blood cell distribution width (RDW), and C-reactive protein (CRP) values were compared between the groups. From routine preoperative laboratory tests, the cut-off values by the receiver operating characteristic (ROC) curve technique were calculated for CRP, neutrophil, and NLR. The cut-off values, which are also compatible with the findings of previous studies, indicate a high probability of conversion [8, 9].

Radiological evaluation

Transabdominal ultrasound (US) was used to evaluate an abnormal appendix as an initial screening method. Two preoperative imaging parameters were analyzed, namely the diameter of the appendix (≤8 millimeters and >8 millimeters), and the US features, including hyperemia, periappendiceal fat inflammation, swollen lymph nodes, free fluid collection, appendicolith, bowel inflammation (terminal ileitis, right-side ileocolitis, pancolitis, non-visualized appendix). In the absence of a visualized appendix on the US, the patients underwent CT scans. The diameter of the appendix is based on the data from the CT in patients with non-visualized appendices in the US.

Surgical procedure

Standard three trocar (umbilical, suprapubic, and lower left quadrant) technique was used in all patients as an initial surgical procedure. The patient was placed in Trendelenburg position and right side up under general anesthesia. Appendix stump was intracorporeally knotted with silk or appendix stump closure was performed using endoloops, hemolog clips, or staplers. The specimens were retrieved through umbilical or suprapubic ports with specimen bags. CA was performed with McBurney's incision or midline laparotomy. In cases of complicated appendicitis, intraperitoneal irrigation was performed with physiological saline solution.

Statistical analysis:

Statistical analyses were performed with NCSS (Number Cruncher Statistical System) software (NCSS, LLC, Kaysville, USA). Descriptive statistical methods (mean, standard deviation, median, frequency, percentage, minimum, maximum) were used when the study data were evaluated. The conformity of quantitative data to normal distribution was analyzed using the Shapiro-Wilk test and graphical tests. Mann-Whitney U test was used for the differences between two groups with quantitative variables with non-normal distribution. The Student’s t-test was used to compare normally distributed continuous data. Pearson Chi-square test, Fisher’s exact test, and Fisher-Freeman-Halton test were used in the comparison of qualitative data. Diagnostic screening tests (sensitivity, specificity, positive predictive value, negative predictive value) and ROC curve analysis were used to determine the cut-off values for the parameters. Statistical significance was accepted as p≤0.05.

## Results

The study was conducted with a total of 521 cases, and the gender distribution for the entire patients was 42.8% (n=223) female and 57.2% (n=298) male. The mean age was 35.17±12.61 years old (range, 16-80 years). The appendectomy was completed laparoscopically in 498 (95.6%) patients, and converted to OA in 23 (4.4%) patients. The mean hospital stay was 2.38±2.13 days (range, 1-21 days). The mean time since the first complaint was 1.79±1.36 (range, 1-10 days). The signs and symptoms of acute appendicitis presented at illness onset varied but pain at the right lower quadrant of the abdomen, and nausea/vomiting were the most prevalent symptoms affecting 492 (94.4%), and 192 (36.8%) patients, respectively. Simple appendicitis was detected in 193 (37%) patients, suppurative appendicitis in 271 (52%) patients, gangrenous appendicitis in 10 (1.9%) patients, perforated appendicitis in 38 (7.3%) patients, carcinoma in 9 (1.7%) patients (Table 1). Furthermore, three of the nine patients with a histopathologic diagnosis of carcinoma required a second surgery due to invasion of the surgical margins.

**Table 1:** The distribution and frequency of the patients’ clinical characteristics and histopathological data

		Mean±SD	Median (Min-Max)
Age		35,17±12,61	33 (16-80)
Hospitalization (day)		2,38±2,13	2 (1-21)
Duration of the symptom onset (day)		1,79±1,36	1 (1-10)
		n	%
Gender	Female	223	42,8
	Male	298	57,2
Groups	Conversion appendectomy	23	4,4
	Laparoscopic appendectomy	498	95,6
Dominant presenting Symptoms	Pain at the right lower quadrant	492	94,4
	Migration of pain	104	19,9
	Food intolerance, Anorexia	60	11,5
	Nausea, Vomiting	192	36,8
	Diarrhea	17	3,2
Histopathological Data	Simple Appendicitis	193	37
	Suppurative Appendicitis	271	52
	Gangrenous Appendicitis	10	1,9
	Perforated Appendicitis	38	7,3
	Carcinoma	9	1,7

The mean age of group 1 was 43.04±15.85 years, and the mean age of group 2 was 34.8±12.34 years. The mean age was significantly higher in group 1 (p=0.012). There was no statistically significant difference between the groups in terms of gender and BMI (p=0.353, p=0.554, respectively). Mean hospital stay after LA was 2.19±1.54 days (range, 1-10 days), while it was 6.48±5.99 days (range, 1-21 days) after CA. The mean hospital stay was found to be significantly longer in group 1 (p=0.001). The mean time since the first complaint was 3.3±2.45 days (range, 1-10 days) in group 1 and 1.71±1.25 days (range, 1-10 days) in group 2. The time from symptom onset to the first examination was significantly longer for patients in group 1 (p=0.001). A statistically significant difference was found between the distributions of the histopathological findings of the cases according to the groups (p=0.001). There was a significant correlation between the histopathologic finding of perforated appendicitis and conversion rates. Pathological findings, operative techniques, clinical characteristics of the patients according to the groups are shown in Table 2.

**Table 2:** Evaluation of descriptive statistics for patients’ clinical characteristics and histopathological data according to the groups ^a^Mann-Whitney U test; ^b^Pearson Chi-square test; ^c^Fisher-Freeman-Halton test; ^d^Fisher’s Exact test *p<0,05; **p<0,01

		Group 1	Group 2	p
Age	Mean±SD	43,04±15,85	34,80±12,34	^d^0,012*
	Median (Min-Max)	42 (18-80)	32 (16-74)	
Gender	Female	12 (52,2)	211 (42,4)	^b^0,353
	Male	11 (47,8)	287 (57,6)	
BMI	Mean±SD	24.6 ± 4.9	24.4 ± 4.8	^a^0.554
	Median (Min-Max)	26.1 (18.75-39.1)	25.8 (18.18- 38.6)	
Hospitalization (day)	Mean±SD	6,48±5,99	2,19±1,54	^d^0,001**
	Median (Min-Max)	5 (1-21)	2 (1-10)	
Duration of the symptom onset (day)	Mean±SD	3,30±2,45	1,71±1,25	^d^0,001**
	Median (Min-Max)	3 (1-10)	1 (1-10)	
Histopathological Data	Simple Appendicitis	5 (21,7)	188 (37,8)	^c^0,001**
	Suppurative Appendicitis	9 (39,1)	262 (52,6)	
	Gangrenous Appendicitis	1 (4,3)	9 (1,8)	
	Perforated Appendicitis	7 (30,4)	31 (6,2)	
	Carcinoma	1 (4,3)	8 (1,6)	

Hyperemia (77.4%) was the most common US finding, followed by periappediceal fat inflammation (35.9%), non-visualized appendix (18.6%), appendicolith (14.2%), free fluid collection (14%), lymphadenopathy (8.4%), and bowel inflammation (4.2%) including terminal ileitis, right-side ileocolitis, pancolitis (Table 3).

**Table 3:** The distribution and frequency of the imaging features

		Mean±SD	Median (Min-Max)
Diameter (mm)		10,13±4,08	9 (1-30)
		n	%
Hyperemia	Yes	403	77,4
	No	118	22,6
Periappediceal fat inflammation	Yes	187	35,9
	No	334	64,1
Lymphadenopathy	Yes	44	8,4
	No	477	91,6
Fluid	Yes	73	14
	No	448	86
Appendicolith	Yes	74	14,2
	No	447	85,8
Bowel inflammation	Yes	22	4,2
	No	499	95,8
Nonvisualized appendix	Yes	97	18,6
	No	424	81,4

There was not a significant correlation between the groups in terms of appendix diameter (p=0.091). A significant correlation was found between the presence of US feature of free fluid collection and conversion rate but no significant difference was found between the groups in terms of other US features (Table 4).

**Table 4:** Evaluation of descriptive statistics for patients’ imaging features according to groups ^b^Pearson Chi-square test; ^d^Fisher’s Exact test; *p<0,05; **p<0,01

		Group 1	Group 2	
		n (%)	n (%)	p
Diameter of the appendix	>8 mm	10 (43,5)	136 (27,3)	^b^0,091
	≤8 mm	13 (56,5)	362 (72,7)	
Dominant ultrasonography features	Hyperemia	14 (60,9)	389 (78,1)	^b^0,053
	Periappediceal fat inflammation	8 (34,8)	179 (35,9)	^b^0,910
	Lymphadenopathy	2 (8,7)	42 (8,4)	^d^1,000
	Fluid	10 (43,5)	63 (12,7)	^d^0,001**
	Appendicolith	2 (8,7)	72 (14,5)	^d^0,758
	Bowel inflammation	1 (4,3)	21 (4,2)	^d^1,000
	Nonvisualized appendix	7 (30,4)	90 (18,1)	^d^0,166

The evaluation of the ROC analysis revealed a statistically significant difference in CRP, NLR, and neutrophil levels between the groups (Table 5).

**Table 5:** The receiver operating characteristic (ROC) curve and diagnostic scan values in each of the CRP, neutrophil and NLR *p<0,05 **p<0,01 PPV: positive predictive value, NPV: negative predictive value, AUC: area under the curve, CRP: c-reactive protein, NLR: neutrophil/lymphocyte ratio

	Cut-off	Sensitivity	Specificity	PPV	NPV	AUC	95% Confidence Interval	p
CRP	≥119	69,57	89,36	23,2	98,5	0,816	0,701-0,932	0,001**
Neutrophil	≥76	82,61	41,57	6,1	98,1	0,636	0,532-0,740	0,027*
NLR	≥6	69,57	57,03	7	97,6	0,643	0,540-0,746	0,020*

**Figure 1:**
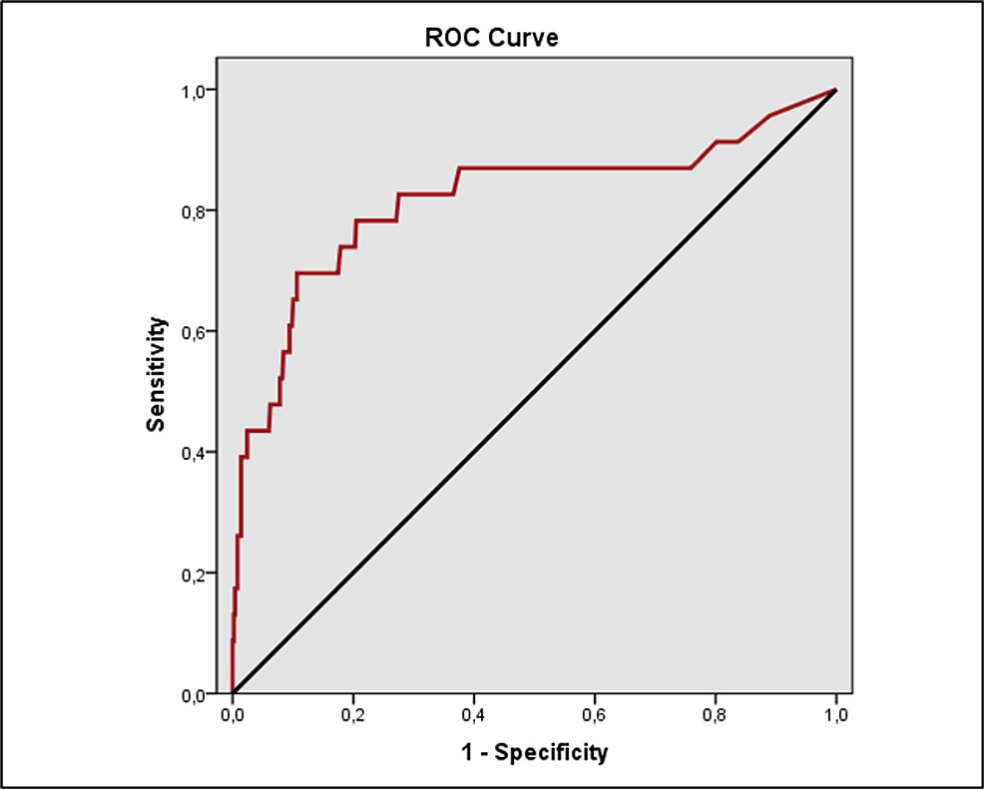
Receiver operating characteristic (ROC) curve analyses of CRP value for the risk of conversion appendectomy in the study group CRP: C-reactive protein

**Figure 2:**
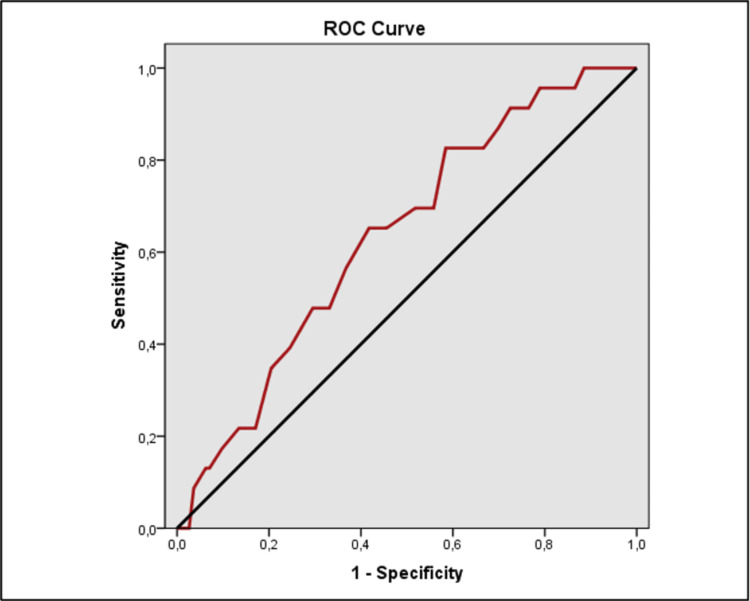
Receiver operating characteristic (ROC) curve analyses of neutrophil value for the risk of conversion appendectomy in the study group

**Figure 3:**
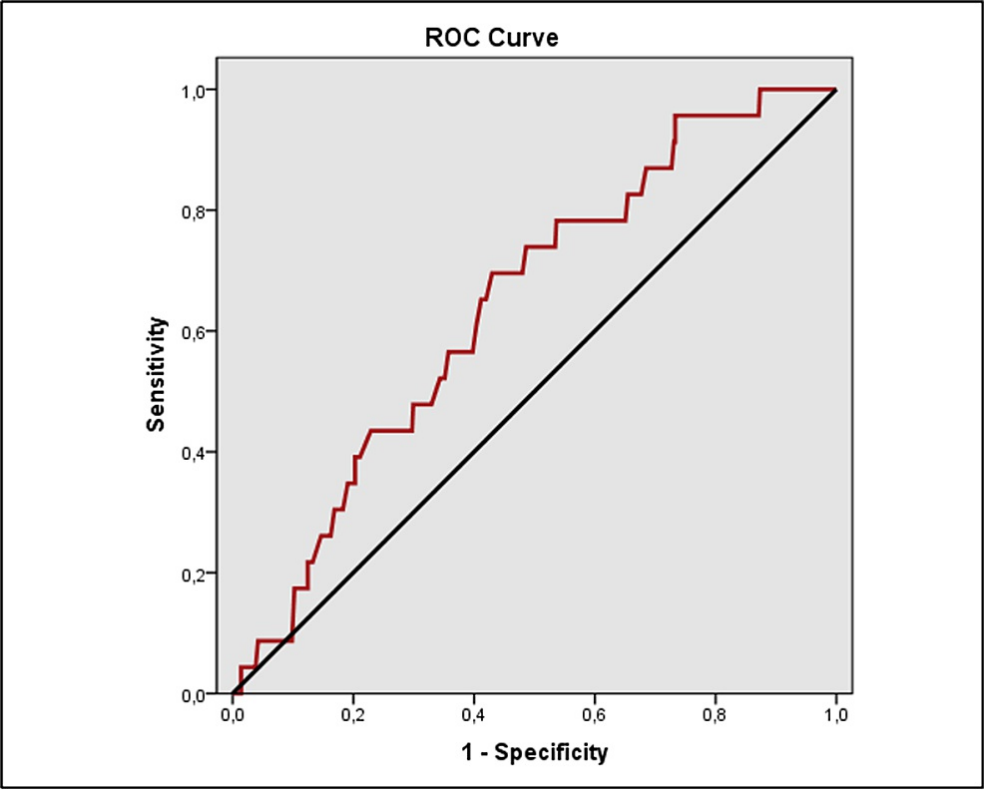
Receiver operating characteristic (ROC) curve analyses of NLR value for the risk of conversion appendectomy in the study group NLR: neutrophil/lymphocyte ratio

ROC analysis found CRP level had a cut-off value of 119 mg/dl to predict the risk of conversion with a sensitivity of 69.57%, specificity of 89.36%, positive predictive value (PPV) of 23.2%, and negative predictive value (NPV) of 98.5%; neutrophil level had a cut-off value of 76% to predict the risk of conversion with a sensitivity of 82.61%, specificity of 41.57%, PPV of 6.1%, and NPV of 98.1%; NLR had a cut-off value of 6 to predict the risk of conversion with a sensitivity of 69.57%, specificity of 57.03%, PPV of 7%, and NPV of 97.6% (Figures 1, 2, 3).

CRP, neutrophil, and NLR levels were identified to be significantly increased (p=0.001, p=0.027, and p=0.02, respectively), and lymphocyte levels were identified to be significantly decreased in group 1 (p=0.026). The mean WBC and RDW levels were 13825.22±4079.96/mm^3^, and 13.04±1.02%, respectively, in group 1. These values were found to be 14100.1±4126.41/mm^3^, and 12.96±1.25%, respectively, in group 2. There was no statistically significant difference regarding levels of both blood parameters (Table 6).

**Table 6:** Evaluation of laboratory values according to patient groups ^a^Mann-Whitney U Test; ^b^Pearson Chi-Square Test; ^d^Fisher’s Exact Test; ^e^Student’s t-test; *p<0,05; **p<0,01 The optimal cut-off values of the CRP, Neutrophil, and NLR were calculated by applying the ROC analysis. CRP: C-reactive protein, WBC: white blood cell, NLR: neutrophil/lymphocyte ratio, RDW: red blood cell distribution width; ROC: receiver operating characteristic

		Group 1	Group 2	p
CRP (mg/dL)	Mean±SD	183,87±142,92	41,59±59,59	^a^0,001**
	Median (Min-Max)	144 (1-534)	15 (1-385)	
	n (%) <119 mg/dL	7 (30,4)	445 (89,4)	^d^0,001**
	≥119 mg/dL	16 (69,6)	53 (10,6)	
WBC (10^3^/µL)	Mean±SD	13825,22±4079,96	14100,10±4126,41	^e^0,755
	Median (Min-Max)	14330 (5810-23330)	13985 (4310-26820)	
Neutrophil (%)	Mean±SD	80,87±6,87	76,12±10,783	^a^0,027*
	Median (Min-Max)	81 (65-91)	78 (6-96)	
	n (%) <76%	4 (17,4)	207 (41,6)	^b^0,021*
	≥76%	19 (82,6)	291 (58,4)	
Lymphocyte (%)	Mean±SD	12,17±5,72	16,13±8,94	^a^0,026*
	Median (Min-Max)	11 (3-26)	15 (2-86)	
NLR	Mean±SD	8,79±6,02	6,82±5,46	^a^0,020*
	Median (Min-Max)	13 (12-16)	13 (11-14)	
	n (%) <6	7 (30,4)	284 (57,0)	^b^0,012*
	≥6	16 (69,6)	214 (43,0)	
RDW (%)	Mean±SD	13,04±1,02	12,96±1,25	^a^0,440
	Median (Min-Max)	7,27 (2,50-30,33)	5,23 (2-86)	

There were various reasons for conversion in group 1. The presence of severe inflammation and adhesions due to inflammation were the most common reasons for conversion (14 of 23 patients, 60.8%). Additional causes of conversion included: location of the appendix (n=2), technical difficulty (n=2), iatrogenic injury (n=1), and other complications (n=4).

## Discussion

Acute appendicitis is the most common cause of acute abdominal pain resulting in surgery in a patient admitted to a hospital [10]. Laparoscopic and open techniques are both preferred in the surgical treatment of acute appendicitis. Gastroenteritis, Meckel's diverticulitis, urinary tract infection, ectopic pregnancy, ovarian cyst rupture, pelvic inflammatory disease, Crohn’s disease, kidney stones are other conditions that can mimic appendicitis [11]. These conditions sometimes cannot be differentiated with physical examination and laboratory tests. The surgeon's first priority is to diagnose life-threatening emergencies. Laparoscopy can be used in the diagnosis of patients with suspected acute abdomen that mimicking acute appendicitis. If there is a doubt about the safety of the patient during laparoscopic surgery, then conversion to open surgery needs to be adopted without hesitation.

Conversion is not uncommon, with reported rates for acute appendicitis between 0% and 47% in previous studies [4]. The steadily rising popularity of laparoscopic surgery and the increase in surgeons' years of experience in laparoscopic applications decreased this rate between 1% and 10% [12, 13]. In the present study, the conversion rate was 4.4% which is lower than those reported previously. The main reason for the low conversion rate in our study was the fact that the cases with a higher likelihood of conversion underwent open surgery as an initial approach. Although laparoscopic surgery has advantages, including reduced blood loss and less postoperative morbidity rates, conversion when necessary is not a setback; it can be a wise choice for the surgeon. Studies have shown that since the complication rates of CA are higher than that of OA, it would be wise to choose open surgery as the first approach in these patients by determining the predictive factors for conversion in advance [6, 14]. Also, it can help prevent long hospital stays and operative times, reduce morbidity, and minimize health care costs.

It is noteworthy that the duration of symptoms was significantly longer for the conversion group. This is in accordance with the previous findings by Gupta et al., who found that symptom duration over two days had a significant impact on the need to convert to open surgery [15]. Different factors leading to conversion to open surgery have been evaluated, and age, male sex, obesity, diabetes, American Society of Anesthesiologists (ASA) scores, duration of symptoms, acute phase reactants, US findings, prior abdominal surgery, dense adhesions, diffuse peritonitis, difficulties in excision of the appendix due to perforation or severe inflammation, technical difficulties, inadequate exposure of appendix, surgeon's experience, intraoperative bleeding, iatrogenic bowel injuries, and other intraoperative complications are found to be associated with increased conversion rates [5, 16, 17]. Similar to the literature, the most common reasons for conversion are the inability to accurately recognize appendix and adhesions as a result of inflammation in this study [18]. A retrospective analysis reported by Wagner et al. evaluated 941 patients and indicated that advanced age, male gender, and obesity were associated with conversion [17]. In our series, whereas advanced age was associated with conversion, elevated BMI and male gender did not predispose a patient to conversion.

The presence of an additional incision in CA increases the need for analgesics and the risk of wound infection, increasing the use of broad-spectrum intravenous antibiotics. These reasons also prolong the hospitalization period of the patients. In this study, it was also reported that the hospital stay was longer in the CA group, which is consistent with the previous studies [19, 20].

It is reasonable to begin with a US in patients with suspected appendicitis. Ultrasonography features like periappendicular free fluid collection, periappendiceal fat inflammation, and appendicolith are commonly related to difficult LA [21, 22]. In this study, the ultrasonographic evaluation revealed that of these features, only the presence of free fluid collection increased the likelihood of conversion to OA. Luminal obstruction is the dominant factor in acute appendicitis either in the form of appendicolith, lymphoid hyperplasia, or parasite infestations [23, 24]. In the present study, 14.2% of the cases operated due to appendicolith. Appendix diameter was not statistically significant in terms of triggering conversion. Similar to the previous report by Goel et al., we may conclude that the increase in diameter on preoperative imaging does not show the high risk of complication or conversion in patients with acute appendicitis [21].

CRP, neutrophil, and NLR levels are reported as the hematological parameters that increase the risk of difficult LA [25-28]. Similar to the literature, we found a strong association between preoperative CRP, neutrophil, NLR values, and high conversion rate. Among studies reporting on the operative difficulty, Ahmad et al. identified high NLR values due to high neutrophils count and low lymphocytes count as a strong predictor for conversion [29]. A study by Shelton et al. also noted that a preoperative CRP level of >150mg/dL is a statistically significant predictor of conversion, corroborates our outcomes [30].

This study indicates that the surgeon should be aware that there is a probability of conversion from LA to OA in the presence of significantly elevated levels of CRP, neutrophil, and NLR, and US finding of free fluid collection. One of the potential weaknesses of this study is that it is limited due to the retrospective nature of data and the heterogeneity of patients. The operations have been performed by different surgeons and the conversion threshold may change in the presence of predictive factors, depending on the individual surgical skills of the surgeons. Also, the study reflects indirect findings and there is a need to have larger studies tend to give more precise reasons for conversion.

## Conclusions

Laparoscopic appendectomy has still a significant rate of conversion to open surgery and conversion is followed by more postoperative complications compared to laparoscopy and open surgery. The severity of the disease is reflected in specific findings from blood tests and imaging studies. In addition, as the severity of the disease increases, the chance of surgery to be completed laparoscopically decreases. It is possible to select patients who may benefit from open appendectomy as an initial surgical approach by identifying predictive factors more precisely.
